# Special Issue: “Advances in Molecular Research on Autoimmune Diseases, 2nd Edition”

**DOI:** 10.3390/ijms27031378

**Published:** 2026-01-30

**Authors:** Davide Cossu

**Affiliations:** 1 Department of Neurology, Juntendo University, Tokyo 1138431, Japan; davide@juntendo.ac.jp; 2 Biomedical Research Core Facilities, Juntendo University, Tokyo 1138431, Japan; 3 Department of Biomedical Sciences, Sassari University, 07100 Sassari, Italy

Autoimmune diseases comprise a heterogeneous group of disorders that are characterized by a breakdown of immune tolerance and the development of aberrant immune responses against self-antigens [1]. Despite their clinical diversity, these conditions share common pathogenic principles, including genetic susceptibility [2,3], environmental and infectious triggers [4], and dysregulated immune signaling pathways [4]. Importantly, autoimmune diseases often involve more than one organ, affecting multiple tissues and systems such as the central and peripheral nervous systems [5], thus substantially impairing quality of life.

Over the past decade, advances in molecular immunology, genetics, and experimental modeling have profoundly reshaped our understanding of autoimmunity [6]. Rather than being driven by a single causative factor, autoimmune diseases are now recognized as the outcome of a dynamic interplay between intrinsic vulnerabilities and extrinsic stimuli [1]. The present Special Issue, “Advances in Molecular Research on Autoimmune Diseases, 2nd Edition”, was conceived to capture this complexity by bringing together original research articles and comprehensive reviews that explore autoimmune pathogenesis from complementary and converging perspectives [7,8,9,10,11].

One of the central themes in this Special Issue is the concept of immune susceptibility as a modulatory, rather than deterministic, state. Baikova et al. [7] provide a compelling example through the development of a transgenic mouse model characterized by elevated circulating levels of cyclophilin A (CypA), a well-known pro-inflammatory mediator. Their findings demonstrate that following an inflammatory challenge, such as dextran sulfate sodium-induced colitis, increased systemic CypA does not induce spontaneous inflammation but does markedly amplify tissue damage [12].

Complementing this concept, the systematic review by Vivó et al. [8] addresses the multifaceted roles of the Goodpasture antigen-binding protein (GPBP), also known as the ceramide transfer protein (CERT). By synthesizing evidence across autoimmune disorders, neurodegenerative diseases, and cancer, the authors of this review emphasize that proteins traditionally assigned to specific cellular functions may exert context-dependent roles across distinct pathological domains [13]. The dual involvement of GPBP/CERT in collagen organization and ceramide transport exemplifies how alterations in extracellular matrix interactions and lipid metabolism may converge and influence immune dysregulation, reinforcing the need for integrative molecular frameworks in autoimmune research [14].

Further evidence from human genetic studies demonstrates that susceptibility loci, identified by genome-wide association studies (GWAS), often converge on immune signaling pathways and metabolic regulators [15,16,17], underscoring the interplay between inherited predisposition and functional molecular networks [18]. Environmental exposures, particularly infectious agents, are increasingly recognized as critical modifiers of autoimmune risk [19,20,21]. In this Special Issue, Ceccarelli et al. [9] provide important clinical evidence supporting this paradigm through an investigation of hepatitis E virus (HEV) infection in patients with systemic and cutaneous lupus erythematosus. Their data reveal a significantly higher HEV seroprevalence in cutaneous lupus compared with systemic disease, suggesting a selective vulnerability linked to tissue-specific immune environments. These findings support the hypothesis that viral infections may not act uniformly across autoimmune phenotypes but instead interact with genetic and tissue-specific factors to shape disease expression [22].

The role of viral triggers is further explored in an experimental setting by Cossu et al. [10], who examine the impact of the Epstein–Barr virus nuclear antigen 1 (EBNA1) in a mouse model of mitochondrial dysfunction. Using PARK2 knockout mice, the authors demonstrate that, in the context of impaired mitochondrial quality control, immunization with EBNA1-derived epitopes is sufficient to induce neuroinflammatory features resembling experimental autoimmune encephalomyelitis. This authors of this study provide mechanistic evidence that viral antigens can drive pathogenic immune responses when mitochondrial dysfunction creates a permissive metabolic and inflammatory environment [23,24].

Beyond genetic susceptibility and environmental triggers, several contributions to this Special Issue underscore the importance of immune–metabolic pathways in regulating the balance between inflammation and tolerance [25]. Stanisavljević et al. [11] focus on dendritic cells, which serve as critical decision-makers at the interface of innate and adaptive immunity. Their study demonstrates that ethyl pyruvate exerts potent tolerogenic effects on dendritic cells by activating the NRF2 antioxidative pathway while simultaneously suppressing NF-κB-driven pro-inflammatory signaling [26,27].

Mitochondrial integrity, ceramide metabolism, and redox regulation are recurrent themes linking multiple contributions within this Special Issue [28,29,30]. Disruption of these pathways is increasingly recognized as a central factor in immune dysregulation, suggesting that metabolic homeostasis is a critical determinant of immune resilience [31]. Therefore, small molecules and targeted therapies that modulate these axes could provide new opportunities for precision interventions in autoimmune diseases [32,33].

Current knowledge gaps include the limited understanding of how systemic metabolic alterations intersect with tissue-specific immune responses [25], the need for refined animal models that better reflect human disease susceptibility [34], and the challenge of translating molecular insights into predictive biomarkers and targeted therapies [35].

Future research should prioritize longitudinal and multi-omic approaches capable of capturing the dynamic interactions between the genetic background, environmental exposures, and immune–metabolic states [36]. Such strategies will be essential for advancing precision medicine in autoimmunity, enabling the identification of individuals at risk, and developing interventions tailored to specific pathogenic mechanisms [37].

The contributions gathered in this Special Issue collectively support a paradigm which discards the idea of autoimmune diseases being isolated immune malfunctions and instead views them as systemic disorders arising from the convergence of genetic susceptibility, environmental and infectious triggers, and dysregulated immune–metabolic networks. By incorporating experimental models, clinical studies, and comprehensive reviews, this Special Issue provides a multidimensional perspective on autoimmunity and highlights promising avenues for future investigation and therapeutic innovation.
